# Feasibility of a novel exercise prehabilitation programme in patients scheduled for elective colorectal surgery: a feasibility randomised controlled trial

**DOI:** 10.1007/s00520-019-05098-0

**Published:** 2019-11-12

**Authors:** Matthew J. Northgraves, Lakshmanan Arunachalam, Leigh A. Madden, Philip Marshall, John E. Hartley, John MacFie, Rebecca V. Vince

**Affiliations:** 1 Hull Health Trials Unit, University of Hull, Hull, UK; 2 Sport, Health and Exercise Science, School of Life Sciences, University of Hull, Hull, UK; 3 General Surgery, Pinderfields Hospital, Wakefield, UK; 4 Department of Biomedical Science, School of Life Sciences, University of Hull, Hull, UK; 5 Academic Surgical Unit, Castle Hill Hospital, Hull, UK; 6 Combined Gastroenterology Research Unit, Scarborough Hospital, Scarborough, UK

**Keywords:** Prehabilitation, Colorectal surgery, Exercise programme

## Abstract

**Background and objectives:**

To investigate the feasibility of delivering a functional exercise-based prehabilitation intervention and its effects on postoperative length of hospital stay, preoperative physical functioning and health-related quality of life in elective colorectal surgery.

**Materials and methods:**

In this randomised controlled feasibility trial, 22 elective colorectal surgery patients were randomly assigned to exercise prehabilitation (*n* = 11) or standard care (*n* = 11). Feasibility of delivering the intervention was assessed based on recruitment and compliance to the intervention. Impact on postoperative length of hospital stay and complications, preoperative physical functioning (timed up and go test, five times sit to stand, stair climb test, handgrip dynamometry and 6-min walk test) and health-related quality of life were also assessed.

**Results:**

Over 42% of patients (84/198) screened were deemed ineligible for prehabilitation due to insufficient time existing prior to scheduled surgery. Of those who were eligible, approximately 18% consented to the trial. Median length of hospital stay was 8 [range 6–27] and 10 [range 5–12] days respectively for the standard care and prehabilitation groups. Patterns towards preoperative improvements for the timed up and go test, stair climb test and 6-min walk test were observed for all participants receiving prehabilitation but not standard care.

**Conclusions:**

Despite prehabilitation appearing to convey positive benefits on physical functioning, short surgical wait times and patient engagement represent major obstacles to implementing exercise prehabilitation programmes in colorectal cancer patients.

**Electronic supplementary material:**

The online version of this article (10.1007/s00520-019-05098-0) contains supplementary material, which is available to authorized users.

## Introduction

Interest continues to grow into preoperative exercise or ‘prehabilitation’ as a strategy to improve postoperative outcomes [1, 2] by increasing physiological reserve in surgical patients [3]. A 2015 Delphi study of 19 consultant colorectal surgeons [4] reported a consensus that exercise programmes should form part of the preoperative care pathway; however, there was no agreement on whether it would be feasible to deliver such interventions within the routine UK care pathway where treatment is required to be initiated with 31 days of the decision to treat [5]. Given the short window that exists for prehabilitation to take place, any strategy employed must be as efficient as possible to achieve worthwhile changes within this timeframe.

Research to date has predominantly adopted either uni-modal cycle-based high-intensity interval training (HIIT) [6, 7] or multimodal interventions incorporating moderate intensity continuous aerobic exercise with more traditional major muscle group targeted resistance exercise and other nutritional and psychological components [8, 9]. These approaches however are dependent on the individual being physically capable of performing such exercises, potentially excluding the less physically able, as well as sufficient preoperative time being available for the intervention to be effective.

With 83% of new cases of colorectal cancer diagnosed in individuals aged 60 years or older [10], many of these patients are likely to have a number of common movement deficits which exist within the general population. For example, age-related reductions in ankle [11] and hip [12] range of motion (ROM) are associated with decreased gait speed, impaired balance and increased risk of falls [11, 13, 14]. Many of these limitations could not only impact on the individual’s ability or willingness to participate in prehabilitation but also affect their ability to perform activities of daily living (ADL). Undergoing major surgery may further exacerbate these deficiencies, increasing the risk of postoperative immobility and decreased postoperative quality of life.

Within a sporting context, it has been suggested that adopting a systematic joint-by-joint approach to functional exercise training may address these issues more effectively than traditional resistance training [15]. Functional training is proposed to target the whole neuromuscular system involved in movement rather than specific single joint and muscle movements [16]. By predominantly adopting weight bearing multi-joint and multi-planar exercises to target joints and muscles involved in providing movement and stability, deficiencies that impact on the body’s ability to act as an efficient kinetic chain can be addressed, improving the ability to perform ADL [15]. Functional prehabilitation training has never been applied to a clinical population; therefore, it may represent a more encompassing format, with the potential to improve preoperative physical functioning and subsequent postoperative outcomes.

The aim of this feasibility trial was to investigate the potential effects of a novel prehabilitation intervention on postoperative recovery. The impact of prehabilitation on preoperative physical functioning and health-related quality of life (HRQOL) were also assessed. Furthermore, the feasibility and acceptability of the intervention to patients within the standard care pathway of NHS secondary care hospital were assessed. This was to inform the potential design of a definitive trial in the future.

## Patients and methods

This prospective feasibility randomised controlled trial (RCT) was conducted in a UK secondary care hospital. Ethical approval was received from the NHS Yorkshire & The Humber NRES Committee (13/YH/0322). The study was registered with clinicaltrials.gov (NCT02264496) and conducted in accordance with the Declaration of Helsinki. Initially adult patients (18 years or older) identified during multidisciplinary team meetings and scheduled for elective colorectal cancer surgery were included. Inclusion criteria were extended to include patients with benign disease after 9 months of recruitment due to the poor recruitment rates. Patients were ineligible if they had a known cardiac or uncontrolled metabolic or respiratory condition precluding exercise, were hypertensive (systolic blood pressure > 180 mmHg and/or diastolic blood pressure > 110 mmHg) and had any pre-existing severe physical disability preventing participation in all components of the prehabiliation programme. However, an inability to perform specific exercises was not a reason for exclusion. The length of prehabilitation was determined by surgical wait time alone, with patients approached once a decision to proceed with surgery had been made, provided a preoperative period of 2 weeks was expected. All participants provided written informed consent to participate.

At entry to the trial, baseline characteristics and details of usual weekly physical activity were collected. The participants completed five physical functioning tests (timed up and go test [TUG], five times sit to stand [FTSTS], stair climb test [SCT], handgrip dynamometry [HGD] and 6-min walk test [6MWT]) and two HRQOL questionnaires (Hospital Anxiety and Depression Scale [HADS] and EORTC Quality of Life Questionnaire-C30 [EORTC QLQ-C30]). The TUG, FTSTS and SCT were performed as previously described [17]. Handgrip dynamometry was alternated between the dominant and non-dominant hand with a total of three repetitions performed per hand. Participants stood in an upright position with their hands positioned by their sides, holding the dynamometer (TKK 5001 Grip A, Takei Scientific Instruments, Shiba, Japan) in their hand. The grip length on the dynamometer was adjusted as required to suit each participant’s hand size, and the setting was documented for the repeat testing. When instructed, participants squeezed for 3 s before relaxing. The 6MWT was used as a measure of functional walking capacity. A single trial was performed in accordance with guidelines outlined by the American Thoracic Society [18] with the exception of a 10-m course being used due to space constraints. Distance walked in metres was recorded at the end of the test. The same standardised instructions were provided for all tests to ensure consistency in performance. With the exception of the 6MWT which was performed once, each test was completed a total of three times following a familiarisation trial with the mean score used for analysis.

Participants were randomised 1:1 to either prehabilitation or standard care using a random number sequence (equal blocks of 20) generated using an online random number generator (www.randomizer.org) with assignments sealed in sequentially numbered opaque envelopes prior to the start of the trial. All assessments were repeated on the evening prior to surgery with the HRQOL questionnaires also posted to participants 3 months after treatment. Length of postoperative hospital stay, postoperative complications and adverse events related to the intervention were recorded along with feasibility based on the number of patients eligible to participate as well as subsequent compliance with the prehabilitation programme.

Participants’ allocated to prehabilitation performed a graded cycling exercise test under medical supervision to volitional exhaustion on an electronically braked cycle ergometer (Ergo Bike Premium, Daum Electronic Gmbh, Furth, Germany) with breath by breath gas exchange collected throughout (Quark B2, Cosmed Srl, Rome, Italy). This was used to aid aerobic exercise prescription. A step protocol was used, starting with 3-min seated rest followed by 2 min at an initial workload of 20 W. This increased by 5–25 W/min until volitional fatigue was reached. Participants were instructed to provide maximal effort with the test continuing until the participant either reached volitional exhaustion, was unable to maintain a cadence of 60 rpm for more than 30 s despite encouragement or they developed any signs or symptoms indicated for the early termination of an exercise test [19].

Participants’ allocated to the standard care were instructed to maintain their normal exercise levels. Participants from both groups completed physical activity diaries during the pre-surgical period to monitor any possible control group contamination.

## Prehabilitation programme

The prehabilitation programme consisted of three individualised 60-min exercise sessions per week delivered on a one-to-one basis by a certified strength and conditioning instructor at the University Sport Science Laboratory. Although the intervention consisted of both aerobic and resistance training (Table 1 and ESI supplementary Table 1), the resistance programme was predominantly based on the principles of functional exercise training [15]. This approach systematically addresses common movement deficits at each major joint complex in the body. These deficits can impair the performance of ADL and were addressed in this intervention using functional exercises performed against either (1) body weight, (2) resistance tubes and bands (Pullum Sports, Luton, UK) or (3) dumbbells (Reebok, Amsterdam, Netherlands) or kettlebells (Eleiko, Halmstad, Sweden) of differing resistance [0.5–10 kg]. Full descriptions of each exercise can be found elsewhere [21]. Starting exercise and resistance were dependent on the participant’s physical capabilities with the programme tailored to the individual. Inability to perform any specific component of the programme was not a reason for exclusion with the intervention adapted to ensure continued participation.

**Table 1:** Outline prehabilitation session structure

Warm up	
5 min of cycle ergometry at 40–50% heart rate reserve (as determined by graded exercise test)	
Resistance circuit 2 (3 to 4 sets)	
Ankle range of motion exercises (seated heel/toe mobilisation, ankle mobilisation, heel walking, sit to stand (with variations in foot placement)	
Medial glute activation (band resisted sit to stand, side lying bent leg hip abduction, X-band walks)	
Thoracic spinal mobility (seated postural exercise variations, standing postural exercise variations, lying thoracic spinal mobilisation with roller (sagittal and transverse), foot raised thoracic extension	
Shoulder function (band pull apart variations, band resisted external rotation, seated row with bands, lying scapular setting)	
Moderate intensity aerobic exercise	
Up to 25 min of cycle ergometry at 40–60% heart rate reserve and/or a perceived exertion of between 11 and 13 on the Borg scale [20]	
Resistance circuit 1 (3 to 4 sets)	
Hip flexor range of motion exercises (standing hip flexor stretch, lying hip flexor stretch, split squat, rear foot elevated split squat)	
Gluteal activation (bilateral lying gluteal bridge, cook hip lift, foot elevated single leg gluteal bridge, shoulders elevated bilateral and unilateral gluteal bridge)	
Whole kinetic chain (kettlebell swings, dumbbell push press)	
Core control (high kneeling band anti-rotation, band resisted side shuffles, suitcase carry, ball passes)	
Cool down	
5-min gentle walking and stretching	

Progressions were applied every two to three sessions, dependent on the participant’s ability to demonstrate correct technique and participant-reported difficulty. Progressions involved either increased repetitions/duration followed by added resistance or progression on to an exercise with greater technical difficulty. Whenever resistance was added, the number of repetitions was reduced to control for training volume. Given the population involved, there were occasions when regression was required for individual sessions (e.g. participant-reported fatigue or muscle soreness at the start of session), and in these instances, repetitions/resistance/duration was reduced accordingly. All progressions aimed to be challenging yet achievable in order to maintain motivation, and encouragement was provided throughout each session.

Initial prescription of aerobic exercise was based on heart rate reserve (HRR) calculated from the graded exercise test. In the first session, 10 min of cycling at 40–60% HRR was performed by participants on an electronically braked cycle ergometer (Ergo Bike Premium). As tolerated, duration of cycling was increased by 2–5 min per session up to a maximum duration of 25 min. Subsequent progressions and regressions in exercise intensity were based on participant heart rate response and/or participant self-reported perceived exertion [20]. Each session was logged by the instructor, detailing attendance, exercises performed (repetitions, sets, duration), measures of intensity (heart rate, perceived exertion) and any components not performed. Daily physical activity during the preoperative period in both groups was recorded through completion of self-report physical activity logs. No specific instructions or restrictions were placed on the physical activity the standard care group could perform.

### Statistical analysis

As a feasibility study, no power calculation was performed for the primary outcome, length of hospital stay. It was proposed that recruitment of 60 patients with full data sets available would provide sufficient information to determine sample size for a definitive study. This estimate was based on the previous work on ‘enhanced recovery after surgery’ protocols [22].

All analyses were performed using IBM SPSS statistics version 20 (SPSS Inc., Chicago, IL) according to an intention-to-treat principle with the primary analysis focused on descriptive statistics. Depending on normality of distribution, either mean ± standard deviation or median and interquartile ranges [IQR] were reported with minimum/maximum values displayed for both.

Between-group comparisons were completed using either independent sample *T* tests or Mann-Whitney *U* tests depending on normality of distribution. Due to the non-normal distribution, within-group changes were assessed using the Wilcoxon rank-sum test with 95% confidence intervals calculated [23].

## Results

### Recruitment

**Fig. 1:**
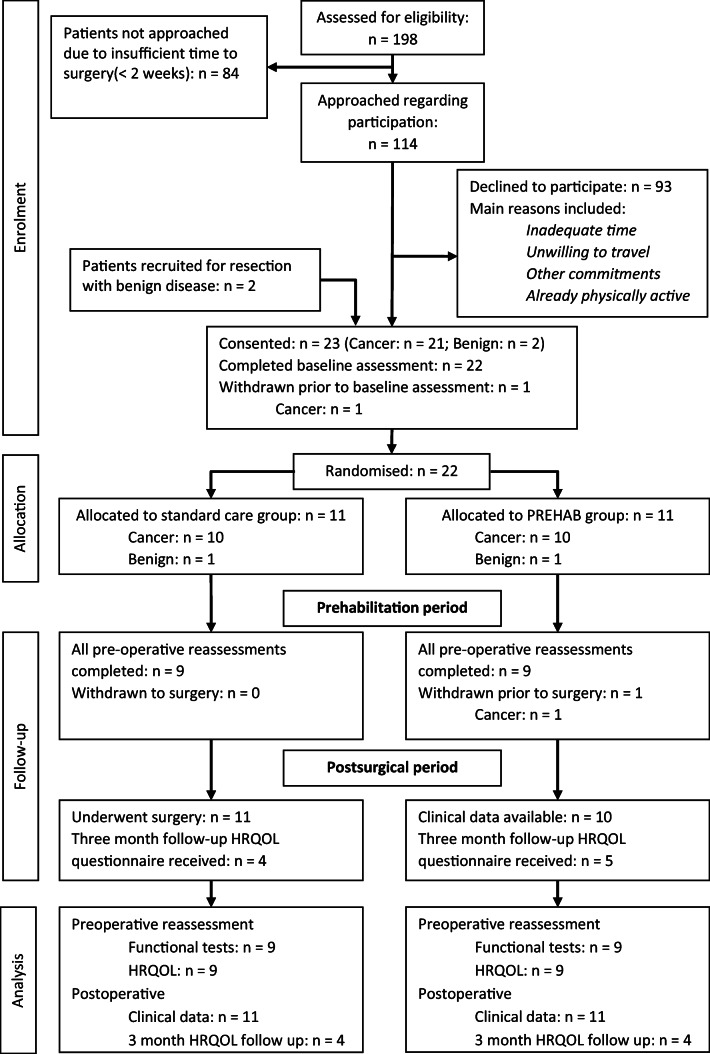
Consort diagram for the study

Within the study, a total of 198 potentially eligible patients were listed for major elective colorectal cancer resection surgery, of which 84 patients (42%) were ineligible due to insufficient time (< 2 weeks) until surgery. Out of the 114 patients approached, 21 colorectal cancer patients consented (18.4%) with two additional patients recruited following the inclusion of patients with benign disease. One cancer patient withdrew prior to baseline assessment whilst one further cancer patient withdrew from the prehabilitation group 2 days prior to surgery having completed the programme, due to an unrelated adverse event. Flow of participants is displayed in Fig. 1 with patient demographics shown in Table 2.

**Table 2:** Descriptive data for patients. Mean ± standard deviation [range]; median (interquartile range) [range]

	Standard care (*n* = 11)	Prehabilitation (*n* = 10)
Age (years)	63.5 ± 12.5 [37; 83]	64.1 ± 10.5 [46; 79]
Gender ratio (M:F)	7:4	4:6
Height (cm)	169.1 ± 11. 9 [147.5; 185.0]	160.9 ± 9.2 [145.5; 176.6]
Body mass (kg)	81.1 ± 25.0 [44.7; 119.0]	78.4 ± 13.1 [57.4; 101.4]
BMI (kg/m^2^)	27.8 ± 5.7 [19.9; 38.1]	30.3 ± 4.3 [24.4; 38.6]
Diagnosis		
Colon cancer	4	3
Rectal cancer	6	6
Diverticular disease	1	1
Surgical approach		
Open	7	6
Laparoscopic	4	4
Neoadjuvant chemoradiotherapy	3	4
Duration between baseline assessment and surgery (days)	16.0 (6) [14; 33]	23.0 (14) [13; 35]

### Acceptability of exercise intervention

In the prehabilitation group, a total of 69 sessions were attended out of a potential 77 (89.6%) by the 10 participants prior to receiving surgery. Individual participant adherence ranged from 75 to 100%, with half (*n* = 5) attending all sessions. Reasons for missing sessions included a pre-arranged holiday (1 session), attendance at family events such as weddings and funerals (3 sessions), work commitments (2 sessions) and transport issues (2 sessions) and were all known in advance. The mean number of sessions attended was 6.9 ± 2.3 [range 3 to 10 sessions] with a mean prehabilitation period of 22.0 ± 7.5 [range 13 to 35] days. A summary of exercises performed across all participants in the prehabilitation group is included in ESI.

### Outcome measures

**Fig. 2:**
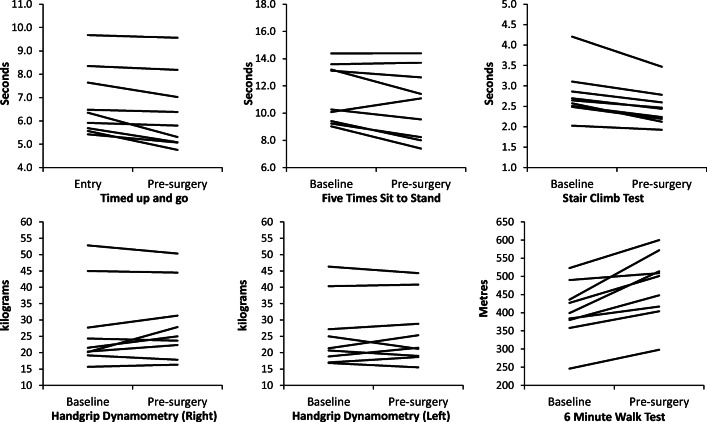
Functional tests—individual performance in the prehabilitation group

**Fig. 3:**
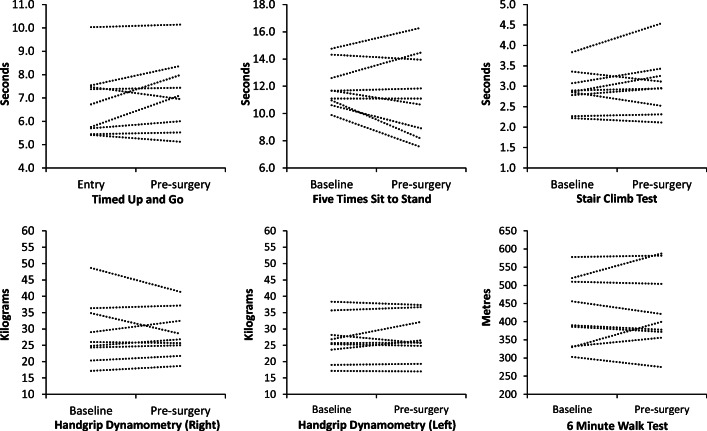
Functional tests—individual performance in the control group

Given the low numbers recruited, the clinical outcomes and HQROL have only been reported descriptively for information (ESI supplementary tables 2 and 3). For the primary outcome, median length of stay was 2 days less in standard care than in the prehabilitation group (8 ± 5 days vs. 10 ± 7 days). All nine prehabilitation participants improved TUG, SCT and 6MWT performance at reassessment (Fig. 2 and Table 3). A similar pattern of change was not evident for standard care (Fig. 3). At reassessment, the distance walked in the 6MWT increased by on average 17.0 ± 9.0% (min/max 3.9 to 31.2%) following prehabilitation compared to the 1.9 ± 10.1% (min/max − 9.2 to 21.2%) following standard care. Similar patterns of change at reassessment were not seen in either group for the FTSTS or handgrip dynamometry.

**Table 3:** Descriptive data for functional tests

		Baseline	Preoperative	Mean/median difference
TUG (s) P	Mean (SD) Median [IQR] Min; Max	6.79 (1.46) 6.35 [2.36] 5.43; 9.68	6.35 (1.63) 5.80 [2.52] 4.76; 9.56	− 0.44 (0.35) − 0.35 [0.60] − 1.04;−0.10
TUG (s) S	Mean (SD) Median [IQR] Min; Max	6.83 (1.49) 6.73 [1.93] 5.42; 10.03	7.18 (1.55) 7.12 [2.42] 5.12; 10.14	0.36 (0.66) 0.11 (1.16) [− 0.52; 1.36]
FTSTS (s) P	Mean (SD) Median [IQR] Min; Max	11.37 (2.16) 10.27 [4.06] 9.03; 14.36	10.71 (2.57) 11.08 [5.05] 7.40; 14.40	− 0.66 (0.92) − 0.37 [2.89] − 2.81; 1.88
FTSTS (s) S	Mean (SD) Median [IQR] Min; Max	11.95 (1.65) 11.67 [2.68] 9.88; 14.76	11.42 (3.01) 10.96 [5.68] 7.52; 16.30	− 0.54 (1.62) − 0.73 [1.59] − 1.79; 1.00
SCT (s) P	Mean (SD) Median [IQR] Min; Max	2.79 (0.61) 2.65 [0.48] 2.03; 4.21	2.47 (0.46) 2.44 [0.53] 1.93; 3.47	− 0.32 (0.18) − 0.27 [0.17] − 0.74; − 0.10
SCT (s) S	Mean (SD) Median [IQR] Min; Max	2.91 (0.50) 2.86 [0.69] 2.22; 3.83	3.02 (0.72) 2.96 [0.93] 2.11; 4.54	0.12 (0.33) 0.05 [0.56] − 0.33; 0.71
HGD, right (kg) P	Mean (SD) Median [IQR] Min; Max	27.4 (12.8) 21.5 [16.7] 15.7; 52.8	28.8 (11.6) 25.0 [17.9] 16.3; 50.3	1.4 (3.2) 0.7 [4.6] − 2.5; 7.7
HGD, right (kg) S	Mean (SD) Median [IQR] Min; Max	29.1 (9.6) 26.0 [13.3] 17.2; 48.7	28.6 (7.3) 26.8 [11.5] 18.7; 41.3	0.45 (3.8) 0.83 [5.1] − 7.3; 3.5
HGD, left (kg) P	Mean (SD) Median [IQR] Min; Max	25.9 (10.5) 21.3 [15.9] 16.8; 46.3	26.1 (10.1) 21.5 [16.0] 15.5; 44.3	0.2 (2.5) 0.5 [4.1] − 3.8; 4.0
HGD, left (kg) S	Mean (SD) Median [IQR] Min; Max	26.6 (6.9) 25.7 [10.6] 17.2; 38.3	27.3 (7.0) 25.8 [12.4] 17.0; 37.3	0.6 (2.3) 0.2 [2.7] − 2.5; 5.3
6MWT (m) P	Mean (SD) Median [IQR] Min; Max	404.8 (80) 399.0 [94] 246; 523	473.7 (93) 501.0 [133] 298; 600	68.9 (37.6) 68.0 [56.5] 19; 136
6MWT (m) S	Mean (SD) Median [IQR] Min; Max	422.8 (97) 390.0 [184] 303; 578	460.7 (106) 400.0 [179] 275; 588	7.9 (38.6) − 6.0 [67.0] − 35; 70

*P* prehabilitation, *S* standard care, *TUG* timed up and go, *FTSTS* five times sit to stand, *SCT* stair climb test, *HGD* handgrip dynamometry, *6MWT* 6-min walk test

## Discussion

In the UK, current NHS targets dictate that treatment in cancer patients is initiated within 31 days of the decision to treat [5] providing only a short window of opportunity to implement prehabilitation, in patients awaiting surgery. In the current study, this has resulted aspects of feasibility and acceptability taking precedent over the original primary outcome. Over 40% of patients assessed were ineligible due to insufficient time existing prior to surgery (< 2 weeks), with the recruitment rate further compounded by a consent rate of 18.4%. This highlights the issue that not only surgical wait time targets present to the feasibility of delivering prehabilitation to elective colorectal cancer patients but also engaging individuals unaccustomed to regular structured exercise programmes presents. Whilst it is important to acknowledge that the availability of a dedicated research nurse may have improved recruitment rates above 18.4%, established multimodal interventions such as cardiac rehabilitation in England only obtain an annual uptake of 50% of those who are eligible [24]. The reasons for non-consent were not formally collected in this study, commonly noted reasons for non-participation included insufficient free time, being unable to travel and other personal commitments. This was despite funding being provided to cover travel expenses and flexible time slots being available to try and promote participation. We speculate that the proposition of undergoing the programme after a recent cancer diagnosis with looming surgery may also have impacted recruitment but also note the high adherence of those who did choose to participate.

In contrast to the majority of literature to date, the intervention in this study adopted the joint-by-joint approach of using individualised functional exercises rather than predominantly focusing on aerobic exercise. This novel approach aims to address common areas of muscular dysfunction and movement impairment, improving movement throughout the body’s whole kinetic chain [15]. This systematic approach aimed to increase mobility in the ankles, hips and thoracic spine, enhance the activation of the gluteus medius and gluteus maximus muscles and improve core and scapulae stability. Positively, although the sample size was limited, the overall acceptability of the intervention for those allocated to the prehabilitation was good, with approximately 90% of sessions attended and all patients completing the invention. Whilst only 5 patients (50%) completed all sessions, over 80% were completed by 9 out of 11 patients. Furthermore, all missed sessions were known in advance and mainly as a result of unavoidable personal events (e.g. weddings, funerals). Although formal qualitative feedback was not collected, general feedback was that it was well received and consistent with previous qualitative work in the field [25, 26].

The predominant focus of prehabilitation in colorectal surgery has been to increase physiological reserve prior to surgery in order to improve clinical outcomes [3]. Understandably, a HIIT-based approach has been advocated [27], given that it has been found to be a safe, well-tolerated and efficient approach to improving objective measures of cardiorespiratory fitness in clinical and non-clinical populations in as little as 4 weeks [6, 28]. From a cardiorespiratory fitness perspective, careful structuring of HIIT interventions according to the patients’ baseline CPET means the exercise intensity should be tolerable in all patients. Positively, in the first trial to demonstrate clinical benefits in abdominal surgery, Barberan-Garcia et al. [27] reported lower postoperative complication rates following a minimum of 4 weeks (mean 6 ± 2 weeks) of prehabilitation. However, two issues exist: (1) Had the minimum of 4 weeks criterion been applied to the current trial, only 3 out of the 21 participants recruited would have been eligible; (2) Despite careful tailoring of the intervention, there is a group of patients who are limited by pre-existing mobility issues or other conditions precluding cycle-based interventions. These patients may still benefit from prehabilitation even when cycle ergometer/treadmill-centred programmes are not suitable. In the current study, two participants were incapable of performing more than 10 min of continuous low-to-moderate intensity (40–60% HRR) cycle ergometer-based exercise and therefore probably would have been excluded from a more cycle ergometer/treadmill-based intervention.

Although the numbers recruited means no conclusive evidence exists regarding this impact of the prehabilitation on physical functioning, a pattern towards improved performance appeared for 3 of the 5 tests (Fig. 2). Whilst the influence performance bias cannot be discounted even in the two patients who could only perform limited amounts of the aerobic component, improvements of 18% and 21% in 6MWT performance were seen. The approach adopted in this programme therefore may allow those who are less physically able or unaccustomed to regular exercise of this type to participate in and benefit from this type of prehabilitation programme. Whether the training load of the programme was enough of a stimulus to illicit a change in aerobic capacity is unknown. However, given that early mobilisation after surgery is an important component of the widely adopted enhanced recovery after surgery approach to surgery [29, 30], a preoperative improvement in physical functioning may translate into a quicker return to normal functioning and improved HRQOL post-surgery.

It is acknowledged that there were limitations to the study that would need addressing if a full trial was to be considered in the future. Firstly, the very low consent rate and overall recruitment rate do represent a potential source of bias in interpreting the results. As a novel prehabilitation programme, in hindsight, the primary outcome of length of stay was unsuitable in this instance. Whilst median length of stay was longer in the prehabilitation group than the standard care group, it is not appropriate to infer this was as a result of prehabilitation given the low sample size and the aforementioned risk of bias. Feasibility outcomes (such as recruitment rate, prehabilitation adherence) would have been a more appropriate primary outcome. Whilst this information has been reported, to ensure transparency of reporting, it is important to report on the pre-specified primary outcome measure. As the tester of the functional tests was not blinded to the group allocation, there is potential for bias to have been introduced, a factor that would be addressed in a definitive trial. The inclusion of the ASA score would have allowed a better grading of functional status at time of recruitment. The inclusion of a qualitative aspect to the study focusing on reasons for non-consent, and patient and care provider perspectives on this approach to prehabilitation and its delivery in the NHS would have been beneficial and should be added to future work.

Future research should further investigate the recruitment pathway (e.g. earlier identification of patients) with more refined inclusion/exclusion criteria. This may include the inclusion of a qualitative aspect focusing on patient and care provider perspectives on this approach to prehabilitation and its delivery in the NHS. A more focused approach on patients who are post neoadjuvant treatment where more time may be available would also be beneficial. Neoadjuvant treatment can cause the deconditioning of the patient [7]; therefore, functional prehabilitation may provide a step towards a more intensive and beneficial prehabilitation programme. Whilst this study focused on an exercise prehabilitation, the inclusion of behavioural change interventions and/or nutritional interventions making it a more multimodal approach to prehabilitation, building on the existing prehabilitation literature [31], would also be advantageous to maximise the protocol and patient benefit.

## Conclusions

Exercise prehabilitation remains an area of interest in preoperative care; however, this feasibility study has highlighted both the logistical issues of short surgical wait times and the challenges promoting patient engagement may present when implemented into the preoperative care pathway. Despite this, with further development, an individual-specific functional exercise-based prehabilitation programme delivered in a group setting may represent a viable alternative approach to HIIT training within ERAS protocols in the future for reducing post-surgery convalescence, especially in those less physically capable patients who may be excluded from more intensive interventions.

## Electronic supplementary material

ESM 1 (PDF 179 kb)
