# Supplementary material for: Feasibility of a novel exercise prehabilitation programme in patients scheduled for elective colorectal surgery: a feasibility randomised controlled trial
Source: Support Care Cancer. 2019 Nov 12;28(7):3197–206. doi: 10.1007/s00520-019-05098-0 (PMC7256075; doi:10.1007/s00520-019-05098-0)
Supplement: Supplementary file 1 — (PDF 179 kb) [file 520_2019_5098_MOESM1_ESM.pdf]

Electronic supplementary information

A novel exercise prehabilitation programme in patients scheduled for elective colorectal surgery: a pilot and feasibility randomised controlled trial.

Matthew J. Northgraves[1,2], Lakshmanan Arunachalam[3], Leigh A. Madden[4], Philip Marshall[2], John E. Hartley[5], John MacFie[6] and Rebecca V. Vince[2]

[1] Hull Health Trials Unit, University of Hull, Hull, UK

[2] Sport, Health and Exercise Science, School of Life Sciences, University of Hull, Hull, UK

[3] General Surgery, Pinderfields Hospital, Wakefield, UK

[4] Department of Biomedical Science, School of Life Sciences, University of Hull, Hull, UK

[5] Academic Surgical Unit, Castle Hill Hospital, Hull, UK

[6] Combined Gastroenterology Research Unit, Scarborough Hospital, Scarborough, UK

	Ankle ROM	Reps	Total Sets	% HRR	Medial gluteal activation	Reps	Total Sets	% HRR	T spine mobility	Reps	Total Sets	% HRR	Shoulder function	Reps	Total Sets	% HRR
Resistance Circuit 1	Seated heel/toe mobilisation	15-20	9	N/R	Band resisted sit to stand	8-12	33	35-45	Seated/standing postural variations	12-15	18	20-25	Lying scapular setting	15-20	8	10-20
	Ankle mobilisation	15-20	156	30-60	Side lying bent leg hip abduction	8-15	84	30-50	Lying t-spine mobilisation	8-12	72	35-45	Band pull aparts	8-12	119	25-65
	Sit to stand	8-12	39	20-45	X-band walks	8-12 steps	90	40-70	Foot raised thoracic extension	8-12	60	50-65	Band resisted external rotations	8-12	51	25-65
													Seated row	8-12	43	30-60
	**Hip flexor ROM**	**Reps**	**Total Sets**	**% HRR**	**Gluteal activation**	**Reps**	**Total Sets**	**% HRR**	**Whole kinetic chain**	**Reps**	**Total Sets**	**% HRR**	**Core control**	**Reps**	**Total Sets**	**% HRR**
Resistance Circuit 2	Standing hip flexor stretch	3 x 15-30 s	62	35-55	Bilateral lying gluteal bridge	8-12	93	30-45	Kettlebell swing	8-15	24	50-90	High kneeling band anti-rotation	8-12	50	40-60
	Lying hip flexor stretch	3 x 15-20 s	7	N/R	Cook hip lift	8-12	34	N/R	Push press	8-12	83	55-80	Band resisted side shuffles	8-12	89	40-80
	Split squat	8-12	65	60-75	Shoulders elevated bilateral gluteal bridge	8-12	9	70					Suitcase carry	10-20 metres	9	N/R
													Ball passes	8-12	55	30-40

%HRR: minimum and maximum value recorded as % of HRR (values do not include all patients; N/R: Heart rate not recorded for this exercise in any patients.
N.B. Values for heel walking, rear foot elevated split squat, foot elevated single leg gluteal bridge & shoulders elevated unilateral gluteal bridge not included as they were not performed

Supplementary Table 1: Combined total number of sets performed and number of reps [range] per set from resistance circuits one & two in the PREHAB group (n = 10).

Supplementary Table 2. Summary of clinical outcomes.

		Standard care (n =11)	Prehabilitation (n =10)	Median difference (95% CI)
**Length of stay**	Median (IQR)	8 (5)	10 (7)	1
	[Min/Max]	[6; 27]	[5; 12]	-3; 6 days
**Patients with complications**		4 (36%)	3 (30%)	
**Adverse events**				
Related		0	0	-
Unrelated		0	1	

Supplementary Table 3. Changes in health related quality of life following prehabilitation or standard care in colorectal surgery .

		**Baseline***	**Pre-operative***	**Change†**
		EORTC QLQ-C30		
PF	P	86.7 [66.7; 100]	93.3 [60; 100]	0.0 [-10.0; 16.7]
	S	100.0 [86.7; 100]	100.0 [53.3; 100]	0.0 [-16.7; 0.0]
RF	P	100.0 [66.7; 100]	100.0 [66.7; 100]	0.0 [0.0; 16.7]
	S	100.0 [83.3; 100]	100.0 [83.3; 100]	0.0 [0.0; 8.3]
EF	P	83.3 [58.3; 100]	75.0 [50; 100]	0.0 [-20.8; 8.3]
	S	100.0 [50; 100]	100.0 [58.3; 100]	0.0 [-8.3; 4.2]
CF	P	83.3 [83.3; 100]	83.3 [83.3; 100]	0.0 [-16.7; 8.3]
	S	100.0 [83.3; 100]	100.0 [83.3; 100]	0.0 [0.0; 8.3]
SF	P	83.3 [33.3; 100]	66.7 [16.7; 100]	0.0 [-16.7; 8.3]
	S	100.0 [66.7; 100]	100.0 [100]	0.0 [0; 16.7]
GHS	P	83.3 [33.3; 91.7]	83.3 [33.3; 100]	8.3 [-8.3; 16.7]
	S	66.7 [16.7; 83.3]	83.3 [33.3; 100]	8.3 [4.2; 16.7]
		HADS		
A	P	4.0 [0; 10]	8.0 [0; 11]	2.0 [0.0; 5.5]
	S	2.0 [0; 7]	3.0 [0; 10]	0.0 [-1.5; 1.5]
D	P	2.0 [0; 7]	2.0 [1; 9]	0.0 [-1.0; 3.5]
	S	2.0 [0; 6]	1.0 [0; 4]	0.0 [-1.0; 0.0]

P: Prehabilitation; S: Standard Care. *Median [min-max]; †Median [95% CI]; PF: Physical functioning; RF: Role functioning; EF: Emotional functioning; CF: Cognitive functioning; Social functioning; GHS: Global health; A: Anxiety; D: Depression.
